# Association between dietary patterns and the prognosis of amyotrophic lateral sclerosis in China: a cross-sectional study

**DOI:** 10.3389/fnut.2024.1437521

**Published:** 2024-10-31

**Authors:** Xun Xu, Yuwei Huang, Yue Zhu, Qingwen Jin

**Affiliations:** Department of Neurology, The Affiliated Sir Run Run Hospital of Nanjing Medical University, Nanjing, China

**Keywords:** amyotrophic lateral sclerosis, special dietary patterns, nutritional status, disease progression rate, prognostic factors

## Abstract

**Background:**

Recently, a growing number of studies have specifically examined the impact of dietary variables on the development and progression of amyotrophic lateral sclerosis (ALS). The purpose of this study was to investigate the correlation between different dietary patterns and Chinese ALS patients’ prognosis.

**Methods:**

A retrospective study was conducted by recruiting 590 patients with ALS who attended and were regularly followed at hospitals in Nanjing from 2016 to 2023. Nutrient intake was calculated using dietary information collected through the food frequency questionnaire (FFQ), and patients were divided into a control group and special diet groups, including a high-calorie group (HC), a high-protein group (HP), and a ketogenic diet group (KD), based on their specific intake. And used the Kaplan-Meier product limiting distribution to compare the time required to transition between phases of different dietary patterns and to estimate cumulative survival probabilities.

**Results:**

Patients in the HP had a better nutritional status. And the disease progression rate (ΔFS) was significantly associated with dietary patterns, with the KD group having the lowest ΔFS. Meanwhile, special diets extended the survival time of stage 4 patients but had no effect on the overall survival of the disease.

**Conclusion:**

A special diet can be one of effective options for patients with advanced ALS. Patients with poor nutritional status may choose the HP diet, whereas those with underlying conditions should consider the ketogenic diet with caution.

## 1 Introduction

Amyotrophic lateral sclerosis (ALS) is a severe neurodegenerative disorder caused by the gradual degeneration of upper (UMNs) and lower motor neurons (LMNs), resulting in the paralysis of voluntary muscles (1). While a minority of patients may experience an extended survival period of up to 10 years, even 20 years, the typical range for the median survival time is between 2 and 5 years (2). The etiology and predisposing factors for ALS remain uncertain. Metabolic disorders and dietary variables have been the focus of significant attention as potential contributors that may lead to faster disease progression and/or accelerated deaths (3). In recent times, there has been a growing emphasis on the impact of nutritional factors on the development and progression of ALS. It has been found that maintaining proper nutritional status during the initial phases of the illness can significantly enhance the quality of life for patients and extend their lifespan (4). Several clinical criteria have been identified as predictors of ALS prognosis, including age and location of disease onset, genotype, clinical phenotype, severity and speed of disease development, level of diagnostic certainty, delay in diagnosis, and cognitive status (5). Nevertheless, the impact of treatment therapies such as riluzole and edaravone (6), enteral nutrition (EN) (7), non-invasive ventilation (NIV) (8), and multi-disciplinary care (9), on survival is still a subject of debate.

There is an increasing interest in investigating how dietary and metabolic factors could influence the prognosis of people with ALS (10–13). Furthermore, several dietary components have been investigated to determine their impact on the development or advancement of the condition, such as vitamins, proteins, antioxidants, and other nutrients, on the progression of the disease, but no conclusive evidence has been found to support either a helpful or detrimental effect (14–17). Possible causes for the discrepancies include insufficient study designs, variations in target demographics, and insufficient statistical power.

The impact of dietary interventions on ALS progression is an emerging area of research. There is a lack of studies that investigate the relationship between various dietary patterns, nutritional status, and disease progression among individuals with ALS. Thus, we conducted a cross-sectional study with a Chinese population to assess the effects of specific dietary patterns on nutritional status and disease progression in ALS patients, with a focus on potential therapeutic benefits.

## 2 Materials and methods

### 2.1 Participants

From September 2016 to September 2023, we conducted a comprehensive analysis of the medical records of all ALS patients who were admitted and consistently monitored at three hospitals in Nanjing, China: The First Affiliated Hospital of Nanjing Medical University, the Affiliated Sir Run Run Hospital of Nanjing Medical University, and the Affiliated Nanjing Hospital of Nanjing University of Traditional Chinese Medicine. We recruited a total of 674 patients who exhibited clinical symptoms indicating probable or definitive ALS, based on the diagnostic criteria recognized as the “El Escorial” criteria (18). Additionally, we enrolled patients who had electrophysiological indications of motor neuron impairment that were in line with the probable or final diagnosis of ALS, as specified in the Awaji-Shima consensus criteria (19). During the course of the study, we found that patients with previous severe dysphagia or PEG were usually on a liquid diet or enteral nutrition, and energy and protein intake were difficult to estimate. At the same time, poor nutritional status and prolonged bed rest made it impossible to record weight changes, and the reduced compliance of such patients posed a challenge for ongoing follow-up. Therefore, we finally excluded patients with severe dysphagia, or PEG, at the beginning.

**Figure 1:**
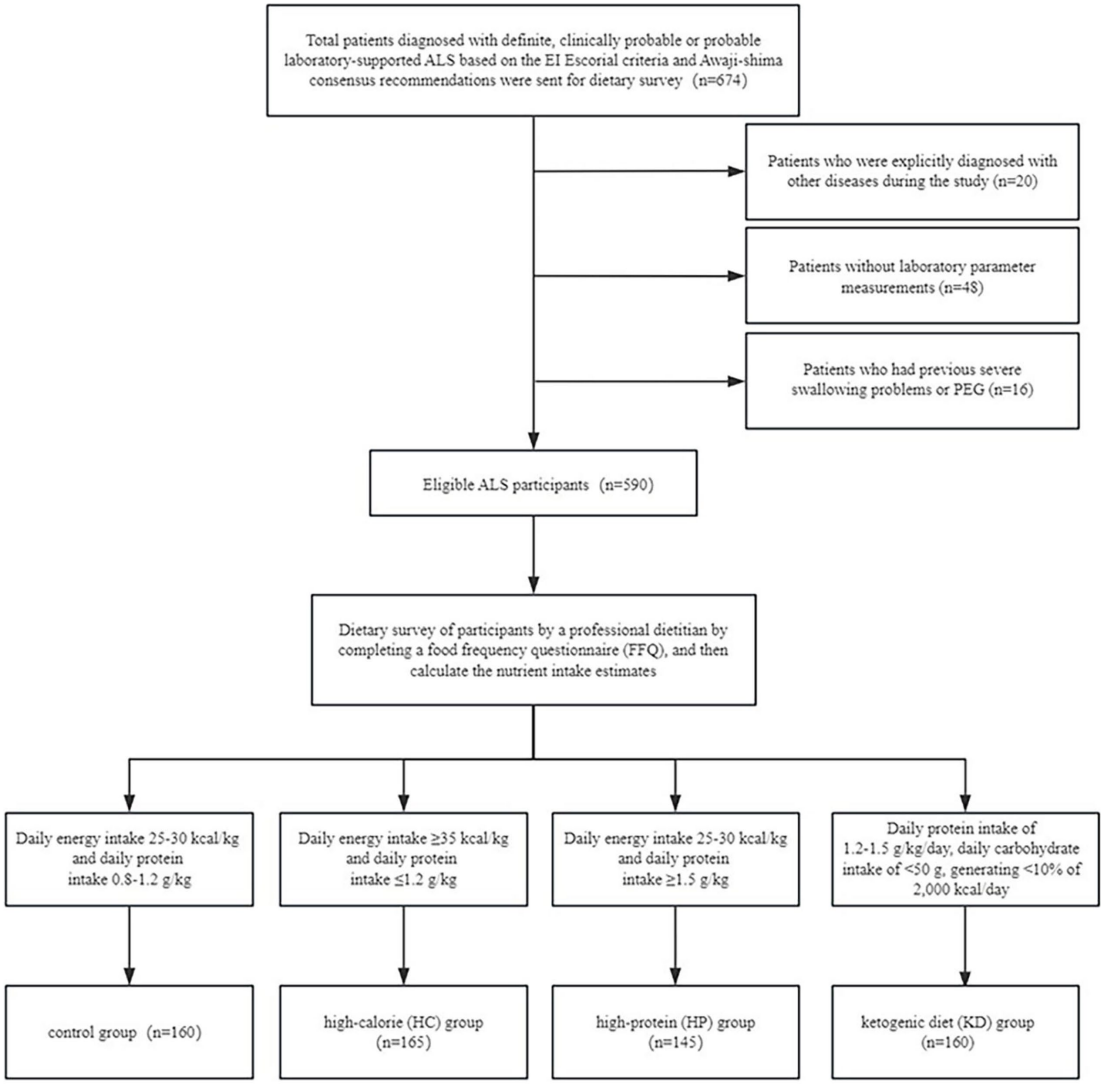
Flow chart for the selection of participants with amyotrophic lateral sclerosis (ALS).

The study excluded patients who satisfied any of the following conditions: (1) patients who failed to submit laboratory parameter measurements (*n* = 48); (2) patients with a history of severe dysphagia or percutaneous endoscopic gastrostomy (PEG) (*n* = 16); and (3) patients who were explicitly diagnosed with other diseases during the study (*n* = 20). This study comprised a total of 590 people who met the eligibility criteria (Figure 1).

The study was carried out in compliance with the Declaration of Helsinki and received approval from the Ethics Committee of the Affiliated Sir Run Run Hospital of Nanjing Medical University (Ethics No. 2022-SR-025).

### 2.2 Data collection

We retrieved the clinical and demographic data of all patients from our medical record database. The acquired data encompassed gender, age at diagnosis, age at onset, anatomical region of disease onset, relevant comorbidities, body weight loss after diagnosis, weight at onset, family history of ALS, ALSFRS-R score (20), and medications used for treatment, cause of death and time of survival from disease onset. At the last known follow-up, survival and other clinical data from living patients were stopped.

Dietary surveys were conducted on participants by a professional dietitian by completing a food frequency questionnaire (FFQ), which has been validated in a healthy Chinese population and has been shown to be reliable and valid for assessing most nutrients and food intake (21). The daily intake of each food item was calculated by considering the average frequency of consumption and the quantity of each food item consumed. The nutrient intake for each food item was determined by multiplying its daily consumption by the nutrient content per 100 grams. And using the Standardized Version of the Chinese Food Composition Table, sixth Edition (Institute of Nutrition and Health, Chinese Center for Disease Control and Prevention, 2018), to calculate the nutrient intake estimates.

Questions related to education level, annual household income, exercise, smoking, alcohol consumption, sports, and sunlight exposure time were also conducted, and the relevant data were analyzed. The definition of exercise required a frequency of once a week or more. ΔFS was determined using the formula (22): ΔFS = (48 − ALSFRS-R score at the time of the survey/duration from symptom onset to the time of the survey (months)). Riluzole and edaravone are the sole drugs approved by the U.S. Food and Drug Administration (FDA) for treating ALS (6).

In addition, all participants enrolled in the study were categorized according to the clinical staging system for ALS proposed by Balendra et al. (23). The lowest clinical stage in this study was stage 2, as there were no patients with enrolment morbidity.

Patients were monitored through regular telephone interviews or clinical visits every 6 months during the trial. At the follow-up, the prognosis, new discoveries, and revisions of the initial diagnosis were documented. During each subsequent visit, a comprehensive assessment of the patient’s symptoms, medical records, death certificates, or any other relevant information was conducted to verify the stage of their illness until their death or until they were no longer being monitored. Participants who survived for 5 years or were lost to follow-up were reviewed at the time of study termination to obtain a final visit, respectively.

### 2.3 Variable grouping

According to the ESPEN Clinical Nutritional Guidelines in Neurology (24), the energy requirements of ALS patients should be calculated at 25–30 kcal/kg/day in the absence of indirect calorimetry. Meanwhile, the Guidelines for the Nutritional Management of ALS (25) suggest that 0.8–1.2 g/kg of protein per day is sufficient to meet the needs of patients with ASL. The ketogenic diet (KD) is a high-fat, protein-rich, low-carbohydrate diet, and there are at least three different versions of the diet (26). The KD consists most notably of moderately high protein (1.2–1.5 g/kg/day) and low-carbohydrate (<50 g/day) intake, generating <10% of 2,000 kcal/day, while fat serves the remaining energy requirement (27). The study further grouped the enrolled participants according to dietary findings: a high-calorie diet (HC) group with a daily energy intake of ≥35 kcal/kg and a daily protein intake of ≤1.2 g/kg; a high-protein diet (HP) group with a daily energy intake of 25–30 kcal/kg and a daily protein intake of ≥1.5 g/kg. The ketogenic diet (KD) group was defined with a daily protein intake of 1.2–1.5 g/kg/day, a carbohydrate intake of <50 g/day, and a caloric intake generating <10% of 2,000 kcal/day, while fat serves the remaining energy requirement. Meanwhile, those with a protein intake of 0.8–1.2 g/kg/day and a calorie intake of 25–30 kcal/kg/day were set as the control group. Following the categorization of food groups, we recommended that patients continue with their existing diets.

### 2.4 Measurement of laboratory parameters

In order to assess laboratory parameters related to nutritional status, participants’ fasting blood biochemical data, and routine blood data were retrospectively analyzed. Biochemical and routine blood data were limited to data from samples not more than two months from the dietary survey. Albumin, creatinine, blood urea nitrogen (BUN), and total cholesterol were measured using an automated biochemical analyzer. Whole blood cells were analyzed using an automated hemocytometer.

### 2.5 Data analysis

The Kolmogorov–Smirnov test was used to examine variables that followed a normal distribution, while nonparametric tests were used to analyze nonparametric variables. The Kruskal–Wallis test or one-way analysis of variance (ANOVA) were used to confirm the continuous variables, which were expressed as means ± standard deviations (SD). Duncan’s multiple range test was then used to confirm the results. The qualitative variables were represented by the number of patients and the percentage of distribution. Differences between the variables were assessed using a chi-square test. The Kaplan–Meier product limit distribution was chosen to compare different dietary patterns in terms of the duration required to transition between stages and to estimate the cumulative survival probabilities. The disparity in the survival curves was assessed using the log-rank test. Cox proportional hazards regression analysis was used to get the risks ratios and 95% confidence intervals after correcting for confounding variables. Covariates (symptom onset age, sex, BMI, site of onset, disease progression rate, drinking, and energy intake) with a *p*-value <0.20 were chosen as confounding factors in multivariate models (28). The statistical analysis was conducted using SPSS (version 27.0; SPSS Inc., Chicago, IL, United States) and PRISM (version 8.0; GraphPad Software, La Jolla, CA). Statistical significance was determined at a *p*-value threshold of <0.05.

## 3 Results

### 3.1 Characteristics of participants according to different diet patterns

Participants in the HP group had a higher blood albumin level compared to the control group, HC group and KD group (Table 1). There were not significant differences among the groups in terms of age at symptom onset, bulbar onset, gender, or symptom duration, education years, annual family income, exercise, sun exposure, smoking, drinking, or treatments received (including riluzole and edaravone), creatinine, urea nitrogen, cholesterol, hemoglobin, TLC and BMI (Tables 1, 2). Participants in the control group had a faster ΔFS than those in the HC group, the HP group and the KD group, and participants in the KD group had the slowest ΔFS. The GNRI score was significantly higher in the HP group than those in the control group, the HC group and the KD group. Compared with other groups, the HP group had the lowest malnutrition rate (86.2%) (Table 2).

**Table 1:** Characteristics of patients with amyotrophic lateral sclerosis (ALS) according different dietary patterns.^1^

Variables	Total (*n* = 590)	Control (*n* = 160)	HC (*n* = 165)	HP (*n* = 145)	KD (*n* = 120)	*p*-value^2^
Sex, male, *n* (%)	370 (62.7)	100 (62.5)	105 (63.6)	85 (58.6)	80 (66.7)	0.939
Age at onset, (y)	54.17 ± 11.77	55.91 ± 11.67	53.91 ± 11.93	51.62 ± 11.12	55.29 ± 12.61	0.520
Onset to diagnosis (mon)	8.47 ± 4.32	7.78 ± 4.18	8.73 ± 4.73	7.79 ± 4.02	9.83 ± 4.14	0.259
Symptom duration (mon)^4^	14.21 ± 9.05	12.31 ± 7.69	13.42 ± 8.68	15.93 ± 9.48	15.75 ± 10.50	0.337
Onset site (bulbar), *n* (%)	130 (22.0)	35 (21.9)	30 (18.2)	35 (24.1)	30 (25.0)	0.934
Education years, *n* (%)						
0–6	165 (28.0)	50 (31.3)	55 (33.3)	25 (17.2)	35 (29.2)	
7–12	270 (45.8)	80 (50.0)	80 (48.5)	60 (41.4)	50 (41.7)	0.414
>12	155 (26.3)	30 (18.8)	30 (18.3)	60 (41.4)	35 (29.2)	
Annual family income, RMB, *n* (%)						
≤5	205 (34.7)	65 (40.6)	70 (42.4)	35 (24.1)	35 (29.2)	
5–10	230 (39)	65 (40.6)	60 (36.4)	55 (37.9)	50 (41.7)	0.585
≥10	155 (26.3)	30 (18.8)	35 (21.2)	55 (37.8)	35 (29.2)	
Exercise^5^, *n* (%)	260 (44.1)	55 (34.4)	65 (39.4)	75 (51.7)	65 (54.2)	0.343
Sun exposure, *n* (%)	365 (61.9)	70 (43.8)	105 (63.6)	95 (65.5)	95 (79.2)	0.054
Smoking, *n* (%)	150 (25.4)	50 (31.3)	35 (21.2)	30 (20.7)	35 (29.2)	0.720
Drinking, *n* (%)	95 (16.1)	35 (21.9)	25 (15.2)	15 (10.3)	20 (16.7)	0.709
Treated with riluzole, *n* (%)	440 (74.6)	120 (75.0)	120 (72.7)	115 (79.3)	85 (70.8)	0.898
Treated with edaravone, *n* (%)	270 (45.8)	45 (28.1)	80 (48.5)	75 (51.7)	70 (58.3)	0.111
Creatinine (μmol/L)	54.91 ± 15.55	53.99 ± 13.52	56.95 ± 21.74	53.78 ± 12.85	54.70 ± 10.78	0.961
Urea nitrogen (mmol/L)	5.25 ± 1.34	5.47 ± 1.19	5.13 ± 1.15	4.95 ± 1.20	5.47 ± 1.82	0.391
Albumin (g/L)	40.01 ± 3.84	39.82 ± 3.03^3^^b^	38.98 ± 3.38^b^	41.91 ± 4.22^a^	39.38 ± 4.31^b^	0.015
Cholesterol (mmol/L)	4.60 ± 0.88	4.73 ± 0.91	4.51 ± 1.01	4.77 ± 0.78	4.37 ± 0.73	0.298
Hemoglobin (g/L)	136.39 ± 14.65	137.03 ± 14.34	137.13 ± 11.30	140.04 ± 16.10	136.26 ± 16.30	0.119
TLC^6^ (×109/L)	1.64 ± 0.55	1.70 ± 0.40	1.57 ± 0.64	1.69 ± 0.47	1.61 ± 0.71	0.611

1 Values are presented as the mean ± SEM or number of patients (percentage distribution), as appropriate.

2 *p*-values were calculated using the Kruskal–Wallis test for creatinine, urea nitrogen, TLC, or chi-square test for qualitative variables.

3 Values with different superscript letters (i.e., a, b) in the same row are significantly different at *p* < 0.05, according to one way ANOVA followed by Duncan’s multiple range test.

4 Symptom duration (months) refers to the time point of the dietary survey from symptom onset.

5 >1 time/week.

6 Total lymphocyte count.

**Table 2:** Disease progression rate and nutritional status of patients with amyotrophic lateral sclerosis (ALS) according to different dietary patterns.^1^

Variables	Control (*n* = 160)	HC (*n* = 165)	HP (*n* = 145)	KD (*n* = 120)	*p*-value^2^
ΔFS	1.59 ± 1.57^3^^a^	1.04 ± 0.84^b^	0.80 ± 0.44^b^	0.78 ± 0.73^b^	0.029
ALSFRS-R score (0–48)	33.47 ± 11.73	37.33 ± 7.73	36.48 ± 7.57	38.75 ± 4.97	0.427
ROADS score (0–56)	37.53 ± 13.95	41.18 ± 10.66	40.90 ± 11.07	44.04 ± 8.25	0.267
BMI (kg/m^2^)^5^	21.96 ± 3.97	21.96 ± 2.88	22.93 ± 2.94	21.20 ± 2.53	0.263
GNRI^6^	100.52 ± 8.86^3^^a^	99.23 ± 7.85^a^	105.48 ± 8.62^b^	98.39 ± 8.08^a^	0.009
No risk, *n* (%)	80 (50.0)	95 (57.6)	125 (86.2)	60 (50.0)	0.017^4^
Low risk, *n* (%)	60 (37.5)	35 (21.2)	10 (6.9)	45 (37.5)	
Moderate, *n* (%)	20 (12.5)	35 (21.2)	10 (6.9)	10 (8.3)	
High risk, *n* (%)	0	0	0	5 (4.2)	

∆FS, disease progression rate; ALSFRS-R, amyotrophic lateral sclerosis functional rating scale-revised.

1 Values are presented as the mean ± SEM or number of patients (percentage distribution), as appropriate.

2 *p*-values were calculated using the Kruskal–Wallis test for ∆FS, ASLFRS-R score, ROADS score, weight or one-way ANOVA for height, BMI, GNRI, followed by Duncan’s multiple range test.

3 Values with different superscript letters (i.e., a, b) in the same row are significantly different at *p* < 0.05, according to one way ANOVA followed by Duncan’s multiple range test.

4 Differences were analyzed using chi-square test at *p* < 0.05.

5 Body mass index was categorized into four groups based on Western Pacific Region of WHO criteria; <18.5, underweight; 18.5 to 22.9, normal; 23 to 24.9, overweight; ≥25, obese.

6 Geriatric nutritional risk index; < 82, high risk; 82 to <92, moderate risk; 92 to 98, low risk; >98, no risk. GNRI was calculated by [1.489 × albumin (g/L)] + [41.7 × (weight/ideal weight)]. Ideal weight was calculated with the equations of Lorentz: height (cm) − 100 − [(height (cm) − 150)/4] for man and height (cm) − 100 − [(height (cm) − 150)/2.5] for woman.

### 3.2 Diet patterns and clinical stages

**Figure 2:**
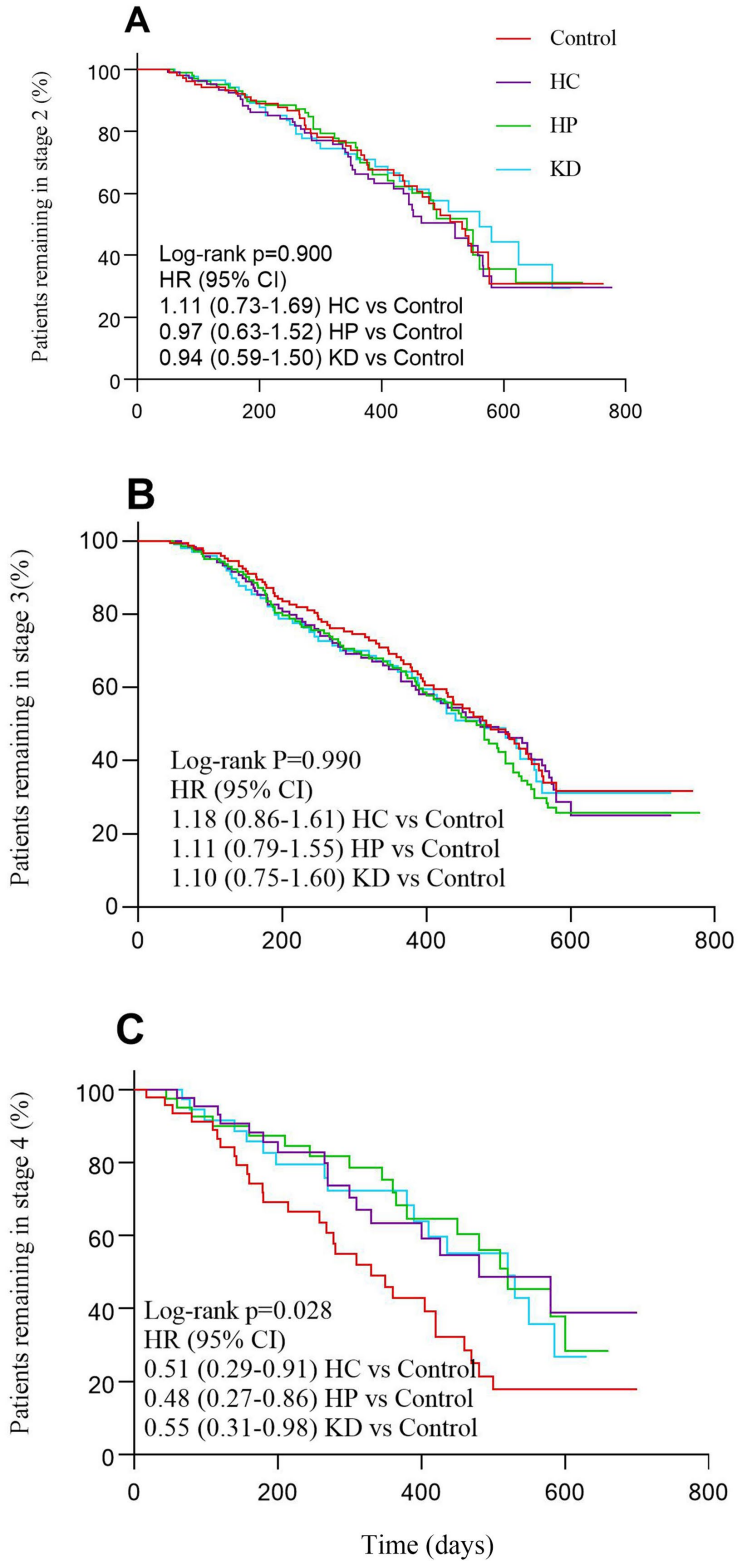
Patients progressing from each stage of amyotrophic lateral sclerosis **(A–C)** according to different dietary patterns.

This analysis revealed that time spent in stage 4 was shorter for patients not transitioning who were in control group than for those not transitioning who were in the HC group, the HC group and the KD group (log-rank *p* = 0.028; Figure 2). Additionally, there were no significant differences in the mean time spent transitioning to a later stage among the different dietary patterns (Table 3). Time from stage 2 (log-rank *p* = 0.900; Figure 2) or stage 3 (log-rank *p* = 0.990; Figure 2) to subsequent stages or death did not differ significantly between control group, HC group, HP group and KD group (Figure 2).

**Table 3:** Stage transition times for patients with amyotrophic lateral sclerosis (ALS) according different dietary patterns.

	Stage 2	Stage 3	Stage 4
Time transitioning to a later stage or death			
Control	354 (172)	358 (184)	270 (176)
HC	346 (171)	363 (190)	323 (170)
HP	337 (164)	362 (190)	357 (179)
KD	333 (168)	330 (176)	341 (178)

Data are mean (SD) times in days. Mean time spent was calculated per patient and averaged over all patients in that group.

### 3.3 Diet patterns and survival time

**Figure 3:**
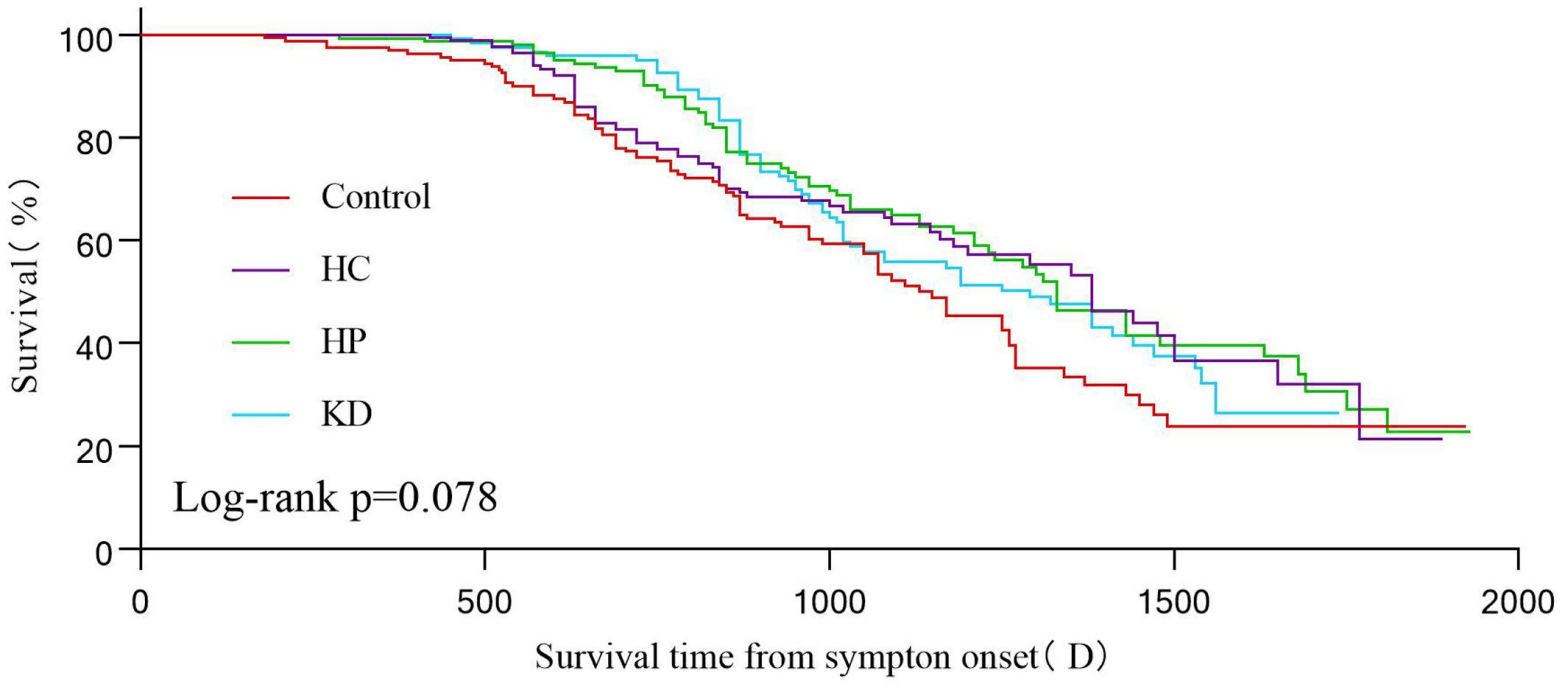
Kaplan–Maier survival curves showing 2000(D) survival according to different dietary patterns. Statistical significance was determined using the log-rank test and the Kaplan–Meier method.

The Kaplan–Meier analysis revealed that there were no statistically significant variations in survival time throughout the follow-up period based on different food patterns (log-rank *p* = 0.078; Figure 3). Nevertheless, our analysis revealed that the overall survival rate in the control group was inferior to that in the HC group, the HC group, and the KD group.

## 4 Discussion

In this study, we conducted a retrospective case-control study on Chinese ALS patients to investigate the relationship between different dietary patterns and disease progression, nutritional status, and survival time of Chinese ALS patients. The findings indicated that the disease progression of ALS patients with special dietary patterns was relatively slow compared with the control group, with the KD group having the slowest disease progression. Meanwhile, it was found that ALS patients on special diets survived longer at stage 4. However, there was no significant difference in overall long-term survival between the groups, which may be related to the relatively small number of stage 4 patients included in the study. Patients with advanced disease may benefit from a special diet. At the same time, this study assessed the nutritional status of patients through GNRI. The results showed that the nutritional status in the control group was significantly worse. Underweight and malnutrition were more common in the control group, while the malnutrition rate among patients in the HP group was lower, and the albumin level was significantly higher than that of patients in other groups.

Creating a suitable ALS metabolic and nutritional support program requires a multispecialty, multidisciplinary approach because ALS patients may have very high demands. Malnutrition can be avoided by implementing a sufficient calorie diet, making dietary adjustments, using adaptive eating gadgets, and installing feeding tubes (25). Nutritional assessment of patients with ALS should be performed at the time of initial referral and continued during follow-up visits to ensure adequate energy and nutrient intake during disease progression (29). Although studies have shown that consuming more foods containing anti-inflammatory and antioxidant compounds is positively associated with reduced oxidative stress in ALS, studies such as those directly comparing the effects of these diets and the mechanisms that lead to ALS and its progression are lacking (30).

Hypermetabolism in the body of ALS patients is one of the most important causes of malnutrition, with an average increase in resting energy expenditure (REE) of 15.9% compared to healthy controls (31). Adequate calorie intake is crucial for ALS patients and therefore ALS patients are encouraged to consume more calories than they calculate (32). A high-calorie diet is an efficient method to stabilize the weight of ALS patients, and high-calorie nutritional interventions could be a new low-cost, low-risk therapy for the treatment of ALS, but there is no conclusive evidence that high-calorie diets prolong survival (11, 33–35). Research using the Gly 86 MAG and Gly 93 ALA superoxide dismutase 1 (SOD 1) mouse models of amyotrophic lateral sclerosis has demonstrated that calorie restriction reduces survival while a high-calorie (36), high-fat diet causes weight gain and delays the disease’s progression (37, 38). A randomized controlled double-blind trial conducted by Ludolph et al. (39) showed that there is no evidence that a high-calorie diet prolongs survival in ALS patients, but improves survival in rapidly progressing patients. In our study patients in HC group gained significantly more weight and had lower malnutrition rate compared to control and KD group patients.

Studies have reported that ALS patients who were supplemented with protein at 1.2 g/kg/day had increased body weight, BMI, and albumin compared to controls, and had more stable ALSFRS-R scores (40). Kim et al. (16) showed that the intake of protein and meat showed a negative correlation with short-term survival and a positive association with survival time in individuals with ALS. This study showed that using the GNRI score to assess the nutritional status of patients, the score of patients on the HP diet was significantly higher than that of patients in other groups, and the HP diet was negatively correlated with malnutrition, but there was no significant improvement in the long-term survival of the patients. Patients with ALS have a reduced muscle mass due to hypermetabolism (41), and an increased intake of protein can stimulate muscle protein synthesis. Studies on high protein intake in critically ill patients have confirmed that high protein intake prolongs survival time and improves the nutritional status of critically ill patients (42, 43). However, there is currently a lack of large studies demonstrating the mechanisms by which protein is beneficial in ALS.

Our current study showed that patients in the KD group had the highest mean ALSFRS-R score (38.75) and the slowest disease progression (ΔFS = 0.75). Zhao et al. (44) tested a ketogenic diet in a SOD1 mouse model, and the experimental evidence suggested that the ketogenic diet slowed down motor deterioration and preserved motor neurons by boosting the energy production of mitochondria in SOD1 mice. The team also found that treatment with caprylic acid, a medium-chain triglyceride that is metabolized into ketone bodies, improved mitochondrial function and neuron numbers in SOD1 mice, but did not improve overall survival (45). At the same time, the ketogenic diet is high in antioxidants and is therefore considered a promising modality for the treatment of neurodegenerative diseases, including ALS (10). Previous research has demonstrated a negative association. Between body weight and BMI and ALSFRS-R scores in ALS patients (46), and while there was no statistically significant distinction in body weight and BMI across the groups in this study, the average body weight and BMI of the patients in the KD group were significantly lower than those in the other groups. This may be due to the fact that KD causes weight loss under the combined effect of reduced caloric intake, increased gluconeogenesis resulting from carbohydrate intake restriction, fatty acid oxidation genes are upregulated while lipid formation genes are downregulated (27). In addition to this, long-term KD may produce a variety of adverse effects, such as anemia, constipation, cardiomyopathy, nausea, vomiting, kidney stones and pancreatitis (47). Therefore, patients with low basal body weight, underlying diseases such as hepatitis, pancreatitis, and anemia should choose ketogenic diet with caution.

This study represents the first retrospective, case-control investigation that explores the correlation between various food patterns and the progression of disease and survival time in patients with ALS. Additionally, it aims to assess the nutritional quality of patients following different dietary patterns. However, this study still has limitations. First, due to a lack of corresponding data, this study cannot draw conclusions about the impact of special diets on ALS onset and stage 1. Second, because this is a retrospective study, subjective factors from patients or family members may bias the findings. Third, despite our promising findings, further in-depth studies are necessary to confirm the role of specific diets in ALS progression, given the limited sample size of later-stage patients. Finally, the sample size of this study mainly came from eastern China, so the relevant research results cannot represent all ALS patients. Large randomized, double-blind, placebo-controlled trials are still necessary to determine the potential therapeutic effects of different dietary interventions on ALS, due to the limitations of retrospective studies.

## 5 Conclusion

Special diets for ALS patients, such as HC, HP, and KD, have a positive effect on improving the nutritional status, especially high-protein diets. However, while special dietary patterns do not significantly improve the cumulative survival rates of patients, they can prolong the survival time of stage 4 ALS patients. Thus, special diets can be one of the effective options to improve the quality of life for advanced ALS patients. In the early stages of the disease, patients can choose whether or not to go on a special diet depending on their condition. If their underlying condition is good, the ketogenic diet may be helpful in slowing the progression of the disease in more rapidly progressing patients. A special diet that includes a high-protein diet may ultimately benefit patients with advanced ALS; however, patients in poor general condition should not follow the ketogenic diet due to its potential side effects. However, due to the limitations of this study, additional clinical trials are required to validate if specific food patterns can improve the prognosis of ALS.

## Data Availability

The data presented in this study are available upon request from the corresponding author. The data are not publicly available due to data management regulations in our hospital.
